# Continuous-variable quantum passive optical network

**DOI:** 10.1038/s41377-024-01633-9

**Published:** 2024-10-16

**Authors:** Adnan A. E. Hajomer, Ivan Derkach, Radim Filip, Ulrik L. Andersen, Vladyslav C. Usenko, Tobias Gehring

**Affiliations:** 1 Center for Macroscopic Quantum States (bigQ), Department of Physics, Technical University of Denmark, 2800 Kongens Lyngby, Denmark; 2 Department of Optics, Faculty of Science, Palacky University, 17. listopadu 12, 771 46 Olomouc, Czech Republic

**Keywords:** Quantum optics, Fibre optics and optical communications

## Abstract

To establish a scalable and secure quantum network, a critical milestone is advancing from basic point-to-point quantum key distribution (QKD) systems to the development of inherently multi-user protocols designed to maximize network capacity. Here, we propose a quantum passive optical network (QPON) protocol based on continuous-variable (CV) systems, particularly the quadrature of the coherent state, which enables deterministic, simultaneous, and high-rate secret key generation among all network users. We implement two protocols with different trust levels assigned to the network users and experimentally demonstrate key generation in a quantum access network with 8 users, each with an 11 km span of access link. Depending on the trust assumptions about the users, we reach 1.5 and 2.1 Mbits/s of total network key generation (or 0.4 and 1.0 Mbits/s with finite-size channels estimation). Demonstrating the potential to expand the network’s capacity to accommodate tens of users at a high rate, our CV-QPON protocols open up new possibilities in establishing low-cost, high-rate, and scalable secure quantum access networks serving as a stepping stone towards a quantum internet.

## Introduction

Quantum key distribution (QKD), the cornerstone of quantum communication, enables two parties to share information-theoretic secure cryptographic keys by exchanging quantum systems over an insecure quantum channel^1^. Currently, QKD is advancing towards commercial applications, forming the backbone of quantum networks through point-to-point (PTP) links with trusted nodes^2–4^.

Recent advancements have also focused on point-to-multipoint (PTMP) QKD connections, addressing the crucial ‘last-mile user access’ problem^5–8^. PTMP QKD using discrete-variable (DV) systems has been proposed for broadcasting channels in passive optical networks (PONs), where a single transmitter is connected to multiple receivers through a passive optical beam splitter^5^. However, the main disadvantage of this configuration is the probabilistic nature of user access and forced time-sharing, i.e., additional privacy amplification is required to compensate for shared user bits provided by the same weak coherent signal pulse^9^. A leading approach to improve network access is based on wavelength division multiplexing that ensures dedicated bandwidth to each user^10–14^. However, the high cost of single photon detectors at each receiver station has limited the applicability of such an approach. As a cost-effective solution, the upstream quantum access network was introduced^6,15–17^, utilizing a time-multiplexing strategy to share a single photon detector among multiple transmitters. However, all aforementioned approaches significantly limit the secret key rate and become increasingly complex with more users due to the time or wavelength slot allocation^18^. Crucially, previous experimental studies have focus primarily on implementation issues using basic PTP QKD connections, while largely neglecting the development of inherently multi-user protocols. Consequently, it is imperative to develop new QKD-based access network protocols that enhance both the secure key rate and overall network capacity, thereby effectively addressing the persistent challenges associated with last-mile user access.

In this article, we propose continuous-variable protocols for quantum passive optical networks (CV-QPON) that facilitate deterministic, simultaneous, and high key rates among all CV-QPON users with information-theoretic security in the presence of Gaussian resources. These protocols extend the scope of CV quantum cryptography from PTP to scalable PTMP networks, which is a crucial aspect for large-scale deployment. We focus on a downstream CV-QPON topology where a provider (Alice) connects to multiple users (Bobs) via an insecure quantum broadcast channel, potentially under adversary control (Eve). Quantum correlations are established by preparing random coherent states at Alice’s station, then simultaneously measured by Bobs. This setup enables independent key generation between Alice and each Bob, thanks to the independent quantum noise experienced by each user and the use of reverse information reconciliation^19^.

Our security analysis encompasses two scenarios: an untrusted protocol, where each Bob views others as potential adversaries, and a trusted protocol, where users collaborate against Eve by relying on a faithful operation of each other. Our trusted protocol uniquely addresses the issue of information leakage due to the residual correlation between users without compromising the secret key’s length. This is achieved by establishing a hierarchical system of trust among users. We demonstrate the feasibility of both protocols through an experimental CV-QPON setup involving eight users, each with an 11 km span of access link. In both trusted and untrusted scenarios, all users can simultaneously generate independent keys secure against collective attacks in the asymptotic regime, with an approximately 30% improvement in the total network key rate for the trusted protocol. Specifically, we achieved a total network key rate of 2.1 Mbits/s (1.5 Mbits/s) for the trusted (untrusted) protocol, and with conservative channel estimation accounting for finite-size effects—1.0 Mbits/s (0.4 Mbits/s). The capacity of our CV-QPON protocol is scalable, allowing it to support more than twice the current number of users, depending on noise level and channel transmittance. Additionally, CV-QPON offers a cost-effective solution as it utilizes standard telecommunications technology, enabling it to be effortlessly integrated into existing access networks.

## Results

### Network architecture and operation

**Fig. 1:**
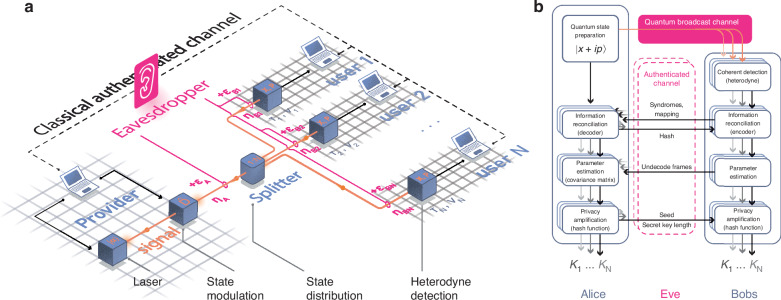
Continuous-variable quantum passive optical network (CV-QPON) architecture and operation. **a** The provider encodes key information onto quadratures of the coherent state. This state is broadcast to *N* users through an insecure quantum channel, under the potential control of Eve. The channel’s properties include transmittance *η* and Eve-induced excess noise, *ϵ*. Users employ coherent detection, decoding key information from the coherent state’s quadrature values (*x* and *p*). The detectors are characterized by quantum efficiency *τ* and electronic noise *v*. All classical communications occur via a classical authenticated channel in a centralized manner. **b** The protocol encompasses a quantum prepare-and-measure phase, followed by data processing. After these steps, Alice and each user will share a symmetric key *K*_*l*_, which can be used for cryptographic tasks

Figure 1a shows the network architecture of CV-QPON, a standard telecom access network topology favored for its high capacity and energy efficiency^20^. Within this network architecture, the nodes are classified according to their distinct roles and functionalities:

- *Provider* (Alice): Generates and randomly modulates quantum states to establish quantum correlations for secret key generation.
- *User* (Bob): Performs heterodyne detection on the received optical mode.
- *Splitter* (1: *N*): A passive component forms a quantum broadcasting channel that connects *N* users to the QPON infrastructure and evenly distributes quantum correlations among them.

In addition, authenticated classical channels are established between the provider and each user. This setup ensures that all classical communication is centralized, i.e., the users cannot communicate among themselves. In the following, we will use both terms ‘Bob (B)’ and ‘the user’ synonymously.

The key generation process in CV-QPON consists of a series of rounds *k* ∈ (1, *M*), each comprised of the following steps:

*Preparation*: Alice draws two random variables *x*(*p*)_*k*_ from independent zero-mean Gaussian distributions ${\mathcal{N}}(0,{V}_{x(p)})$ to encode information into a coherent signal state by means of the modulation process. The quadrature variance of the generated state is 1 + *V*_*x*(*p*)_. The preparation station is assumed to be trusted, meaning that it neither leaks information to an eavesdropper nor allows an eavesdropper to control noise within the station.

*Distribution*: The quantum states are transmitted through an untrusted quantum channel, fully controlled by an eavesdropper with transmittance *η*_*A*_, to a splitter, where the coherent signal state is divided and sent to each user through individual untrusted quantum channels with transmittances ${\eta }_{{B}_{l}}$. The channel is modeled by passive linear optical elements, and the total transmittance is given by ${\eta }_{l}={\eta }_{A}{\eta }_{{B}_{l}}/N$. Here, we distinguish between total transmittance *η*_*l*_, which covers the entire link, and the segment-specific channel transmittance *η*_*A*_*η*_*B*_. Each link subjects the quantum states to varying levels of noise, with the total excess noise received by each user given by ${\varepsilon }_{l}={\varepsilon }_{A}{\eta }_{{B}_{l}}/N+{\varepsilon }_{{B}_{l}}$.

*Detection*: Each user measures the incoming quantum states, monitoring the level of electronic noise (with quadrature variance *ν*_*l*_) and detection efficiency *τ*_*l*_.

*Post-processing*: After *M* rounds, Alice engages in data processing with each user over authenticated classical channels, as depicted in Fig. 1b. This includes information reconciliation, parameter estimation, and privacy amplification. Unlike PTP settings, where each round *k* is dedicated to a single user *l*, in CV-QPON protocols, Alice processes the data in parallel by replicating sequences *x*(*p*)_1,⋯,*M*_ to generate *N* independent secret keys.

In the CV-QPON, it is also possible to split the quantum states into *N* unequal parts. This allows prioritization of certain users or meeting demands of larger keys for preferred services. Nonetheless, the primary focus of this work is on maximizing the number of users that can be supported simultaneously while adhering to the principles of net neutrality. The following section will delve into the protocols that can be implemented within CV-QPON, particularly those facilitating simultaneous key establishment between Alice and each Bob.

### Basic CV-QPON protocols

The asymptotic key rate is deemed to be secure if the lower bound on the difference between the mutual information of trusted parties ${I}_{A{B}_{l}}$, and accessible information of Eve on measurement of the reference side ${\chi }_{E{B}_{l}}$ remains positive^21^:

1 $${K}_{l}(\eta ,\varepsilon )=\max \left[0,{\beta }_{l}{I}_{A{B}_{l}}-{\chi }_{E{B}_{l}}\right],$$

where *η* and *ε* are channel parameters, *β*_*l*_ is the efficiency of information reconciliation. Both mutual information ${I}_{A{B}_{l}}$ and Holevo bound ${\chi }_{E{B}_{l}}$ are determined by the covariance matrix of the overall shared multipartite state. For simplicity of notation, we omit detection efficiency and electronic noise. However, they are incorporated in the respective covariance matrices and security analysis. For further details see Supplementary materials.

#### Time-sharing approach

The most basic method to manage network access among users is known as time-sharing^6^. In this approach, each round *k* is allocated to a specific user *l*. However, the time-sharing PTP QKD protocol faces a significant limitation in key rate as the number of users in the network increases. This is because only a fraction of the rounds, specifically *M*/*N* rounds, are designated for a key generation for each user. Under the assumption that all links between the splitter and users have the same losses ${\eta }_{{B}_{l}}={\eta }_{B}$ and noise ${\varepsilon }_{{B}_{l}}={\varepsilon }_{B}$, the *total secret key rate* generated within the network can be expressed as

2 $${K}_{\Sigma }^{{\rm {TS}}}=K({\eta }_{l},{\varepsilon }_{l}),$$

which is equal to the standard PTP key rate with a single user over a channel with parameters *η*_*l*_, *ε*_*l*_^22^. The time-sharing protocol is particularly suitable for DV QKD-based access networks, where the key is generated by single-photon signal states, and all users time-share the single-photon detector^6^. However, CV coherent states, whose amplitudes can be split into different modes, enable *simultaneous* and *deterministic* key distribution among different users, and these advantages are utilized in the following broadcasting protocols.

#### Untrusted broadcast protocol

Due to the multiphoton nature of the coherent state and the use of coherent detection in CV-QPON, detection events will occur for all users in each round of the protocol. Despite the broadcasting of the same coherent state across the CV-QPON, each user, after *M* rounds, obtains measurement outcomes that are unique, yet weakly correlated. This uniqueness arises from independent quantum noises affecting each user differently. Through the application of reverse reconciliation^19^, Alice can concurrently generate *N* keys using the measurement result of each user as a reference (a related idea has been introduced in ref.^5^). After undergoing the privacy amplification process, these keys become completely independent^23^. It is critical to ensure that the cost of privacy amplification is sufficient to decouple the final key *K*_*l*_ from Eve *and* all other users.

To assure the key independence within the network, one can assume that the fraction, (*N*−1)/*N*, of the split signal is intercepted by Eve, instead of being distributed to (*N*−1) users. This necessitates that each user operates under the presumption that other users may collaborate with Eve. By adopting this assumption, an upper bound can be established on Eve’s information. Consequently, under this framework, the total network key rate is quantitatively defined as:

3 $${K}_{\Sigma }^{U}={\sum \limits_{l=1}^{N}}{K}_{l}=N\times K({\eta }_{l},{\varepsilon }_{l}).$$

This approach invariably offers an advantage over the time-sharing protocol as all *M* rounds are designated for key generation. The concept of this untrusted protocol was theore tically explored in ref.^23^. However, this study made a specific assumption about the scaling down of channel-related excess noise with an increase in the number of users, thereby overestimating the network’s capacity.

### Advanced CV-QPON protocols

In the following section, we outline an issue of excessive trust within the network and introduce a protocol that takes advantage of the multi-user nature of the broadcasting protocol and benefits from the dependable operation of network users without jeopardizing the security.

#### Improving network performance

In PTP CV-QKD protocols, the security level can be defined based on the degree of trust assigned to different parts of the system, specifically, those parts that can potentially be under/beyond Eve’s control^24,25^. Typically, a higher security level implies fewer assumptions about Eve’s ability to access and control the system. This, in turn, influences both the achievable key rate and the secure distance that can be reached. However, some deviations from nominal performance, e.g., imperfect detection, including non-unity quantum efficiency and electronic noise, can be regarded as trusted, provided that the respective equipment is thoroughly characterized and monitored. These deviations then do not enhance Eve’s knowledge about the key.

In this work, we extend this notion of trust among QPON users. Specifically, when user *B*_*i*_ trusts user *B*_*j*_, *B*_*i*_ assumes that *B*_*j*_ successfully receives and measures the 1/*N* portion of the signal, instead of it being intercepted by Eve. This shift in perspective enables *B*_*i*_ to attribute the corresponding signal loss to an overall trusted multipartite state, rather than to Eve’s intervention. Consequently, this lowers the accessible information of Eve ${\chi }_{E{B}_{i}}$ while maintaining the mutual information between the provider and *B*_*i*_, denoted as ${I}_{A{B}_{i}}$. Thus, it enhances the overall key rate.

To obtain ${\chi }_{E{B}_{i}}$, it is necessary to reconstruct a covariance matrix corresponding to the trusted state, which contains modes of Alice, Bob_*i*_ and Bob_*j*_, along with the purifications of realistic detectors^26^. The reconstruction of this matrix involves estimating parameters for both users (*η*_*i*_, *ε*_*i*_, *τ*_*i*_, *ν*_*i*_ and *η*_*j*_, *ε*_*j*_, *τ*_*j*_, *ν*_*j*_), a task performed by Alice. For a detailed description of the modeling of this trusted system, please refer to Supplementary material.

On the other hand, misplaced assumptions can undermine the security of the entire network. Suppose all users have full trust in each other’s faithful operation. In an attempt to establish keys, Alice reconstructs a full covariance matrix with *N* users. She presumes that Eve can only access information from ancillary modes before and after the splitter with a total number of modes equal to *N* + 1. However, during information reconciliation, each user transmits a syndrome related to their measured data, as shown in Fig. 1b. This allows Alice to reconcile her data string based on the reference user’s measurement. Since all users are correlated, every syndrome provides non-negligible information about non-reference user’s measurements as well. This issue is further amplified by the inefficiency of reconciliation algorithms *β* ∈ [0, 1), necessitating sending a larger syndrome than the theoretically required minimum. Hence, if all users simultaneously attempt to minimize the cost of privacy amplification, they might significantly underestimate Eve’s information, thereby endangering the network’s security.

One way to solve this issue is to use part of the generated key from the previous QKD session to encrypt the syndrome with a one-time pad^27^. However, the exact encryption cost in terms of reserved key volume that would be sufficient to preserve the security must be determined in advance. Additionally, the amount of pre-shared key needed to initiate the protocol also increases significantly. Furthermore, the irreversible property of the protocol, i.e., each QKD session is independent of the others, no longer holds in this context.

#### Trusted broadcast protocol

**Fig. 2:**
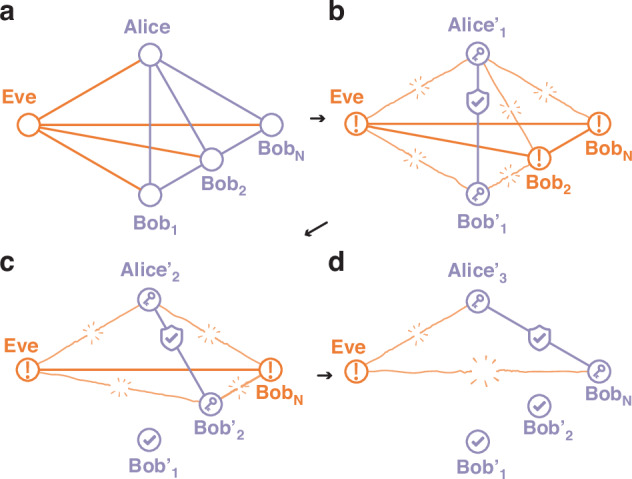
Correlations between different parties in broadcasting protocols. **a** Correlations after signal broadcasting. **b** Alice_1_ and Bob_1_ do not trust other users and decorrelate their joint data from everyone else, resulting in the unique decoupled identical secret key at Alice${\prime}$ and Bob${\prime}$. In the untrusted broadcasting protocol, all users perform only this step. **c** Alice_2_ and Bob_2_ now are decorrelated from Alice${\prime}$ and Bob${\prime}$, and hence, consider them trusted. **d** Alice_*N*_ and Bob_*N*_ trust previous pairs to decorrelate their data from them, thus can pay the lowest privacy amplification cost

We introduce a new protocol that outperforms untrusted protocols in terms of network key rate while avoiding disclosing information regarding other network users through the syndromes. Upon completing *M* rounds of the protocol, Alice initiates key distillation with multiple users simultaneously. Starting with *B*_1_, who considers *B*_2_ ⋯ *B*_*N*_ as *untrusted* parties, effectively under Eve’s control, he opts for the maximum privacy amplification penalty. However, this also implies that no other Bob can threaten the security of the final key, *K*_1_. Knowing this, *B*_2_ can now classify *B*_1_ as a trusted user, as there is no threat to the security of *K*_1_ from his actions, though he still regards *B*_3_ ⋯ *B*_*N*_ as untrusted users. This strategy enhances the secure key rate, as in a simplified scenario with identical parameters in all *N* channels, *K*_1_ < *K*_2_. Following this pattern, as illustrated in Fig. 2, each successive user trusts all preceding users, accruing an additional key advantage progressively. By varying the order of trust among users in each session, we can optimize the network key rate gain ${K}_{\Sigma }^{{\rm {T}}}\ge {K}_{\Sigma }^{{\rm {U}}}$, where equality holds only when no key can be established, without violating the security of any individual user.

**Fig. 3:**
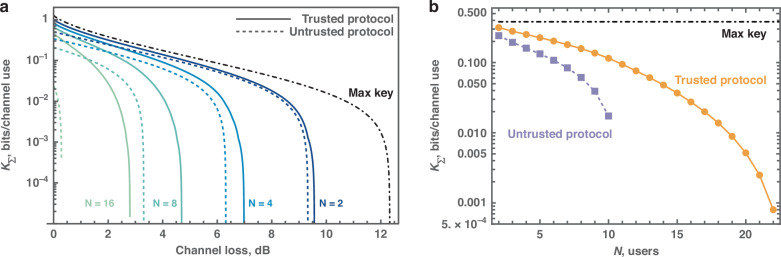
Performance and capacity analysis of CV-QPON protocols. Comparison of the theoretical performance of the untrusted protocol (dashed), trusted protocol (solid) and maximal achievable key (dot-dashed) in terms of the lower bound on the total network key rate *K*_*Σ*_ depending on **a** the channel loss *η*_*A*_*η*_*B*_ with different numbers of users *N* = 2, 4, 8, 16. **b** Number of connected users at fixed channel loss *η*_*A*_*η*_*B*_ = −2 dB. Parameters: reconciliation efficiency *β* = 95%^29,30^, modulation variance *V*_*x*(*p*)_ = 4 SNU (which is optimal for a large number of users), detector efficiency *τ* = 86%, electronic noise *ν* = 2% shot-noise unit (SNU), excess noise at channel output *ε* = 0.5% SNU

In scenarios where certain users are unable to generate keys, it does not necessarily indicate a comprehensive compromise of the network’s ability to generate secret keys with those users. Indeed, they can still attain a positive key rate by adopting a greater degree of trust. Figure 3 delineates this principle, showing that under conditions of higher loss or an increased number of users, the untrusted broadcast protocol fails to maintain a non-zero key rate. In contrast, the trusted protocol continues to yield a positive key rate for some users even under these challenging conditions. Furthermore, Fig. 3 provides a comparative analysis of the performance of two broadcast protocols against the maximal key rate achievable through a PTP protocol. This analysis is conducted under identical conditions of channel loss, *η*_*A*_*η*_*B*_, and equivalent levels of excess noise, *ε*, at the output of the quantum channel. The comparison indicates the possibility of an optimal CV-QPON protocol capable of further improving the total network key or even saturating the PTP key rate. Notably, the larger the network, the greater the quantitative improvement of the key rate when users are assumed to be trusted.

### Experimental investigation

**Fig. 4:**
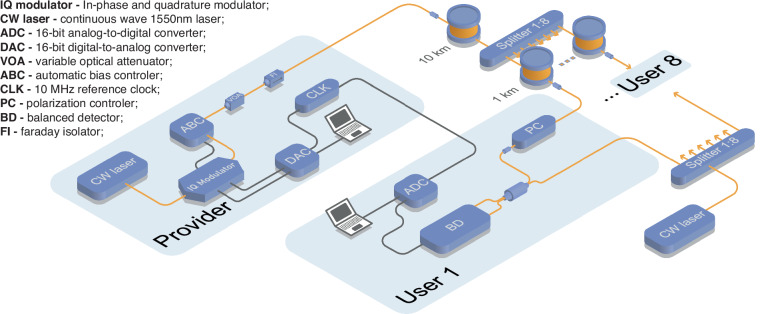
Experimental setup of CV-QPON. The setup involves a provider, Alice, generating a coherent state in the single sideband of the optical carrier, utilizing a CW laser and an IQ modulator driven by a DAC and an automatic ABC. The system connects Alice to eight users via a passive optical splitter and fiber spools. Each user employs RF heterodyne detection, utilizing an independent CW laser as an LO shared among users, a BD, and a PC to adjust the quantum signal’s polarization. The detected signals are then digitized using a 1 GSample/s DAC card, synchronized to the DAC with a CLK

Figure 4 shows the schematic of a CV-QPON network consisting of a provider connected to eight users through a quantum channel composed of a 1:8 beamsplitter and 11 km of standard single-mode fiber. The provider generates a coherent state at a symbol rate of 100 MBaud in the single sideband of the optical carrier using a continuous wave laser, in-phase and quadrature modulator, and an automatic bias controller, while each user detects the quantum information using RF heterodyne detection (see the “Materials and methods” section for a detailed description of the experimental implementation).

In our network architecture, we implemented a local LO (LLO) scheme to rule out side-channel vulnerabilities and simplify the network setup. A main challenge encountered with this approach is the laser phase noise, mainly arising from the use of independent lasers at the provider and user stations. Although all users share the same LO, we assume that the excess noise affecting each user is independent. This is because each user has a separate fiber channel, and each channel introduces its own independent phase noise. However, in general, noise correlations could influence key rate performance depending on other network parameters.

**Fig. 5:**
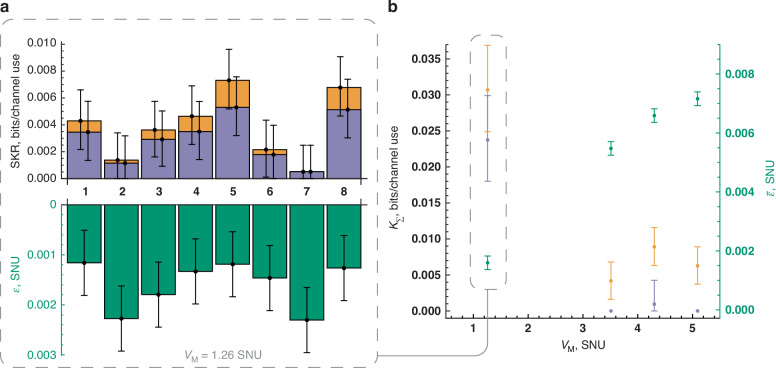
Performance of CV-QPON protocols—experimental results. **a** The top section illustrates the secure key rate (SKR) achieved by each user with a modulation variance of *V*_M_ = 1.26 SNU for the untrusted protocol (violet bars) and trusted protocol (orange bars), with an assumed reconciliation efficiency of *β* = 95%. The bottom section displays the corresponding excess noise measured at the user stations. Noise error bars are determined by Gaussian confidence intervals with 6.5 standard deviations and a failure probability of *δ* = 10^−10^ ^31^. SKR error bars are given by respective lowest/highest noises and transmittances within confidence intervals. **b** Total network key rate *K*_*Σ*_ and mean excess noise $\bar{\varepsilon }$ for all eight users at different modulation variance settings

Optimizing the modulation variance, *V*_M_, for a specific reconciliation efficiency, *β*, can theoretically enhance protocol performance. However, in practice, increasing *V*_M_ leads to higher excess noise, attributable to the residual phase noise. This correlation is evident from the linear scaling of the excess noise with *V*_M_, as highlighted in Fig. 5b. Consequently, this complicates the determination of an optimal range for *V*_M_^28^ for a given *β*. Thus, to facilitate concurrent network access, we chose a *V*_M_ of 1.26 shot-noise units (SNU) to align with both the MET-LDPC code rate and the levels of expected excess noise.

Figure 5 compares the total key rates of both trusted and untrusted CV-QPON protocols at various modulation variances, respective measured mean excess noise $\bar{\varepsilon }$, with a *β* of 95%^29,30^. It shows that minor alterations in channel parameters can significantly impact key rates, underlining the importance of carefully managing modulation variance to support simultaneous key generation for all users. Compared to the untrusted protocol, our developed trusted protocol (indicated by orange bars) increases the total network key rate by ≈30% and enables a positive key rate at higher *V*_M_, where phase noise is more pronounced.

Table 1 summarizes the experimental parameters for key generation, covering the full protocol implementation, including information reconciliation and privacy amplification. Alice generated *M* = 10^8^ coherent states with a modulation variance of *V*_M_ = 1.26 SNU. The trusted loss at the user stations was *τ* = 0.685. Information reconciliation yielded various efficiencies *β* and frame error rates (FER), reflecting the different received signal-to-noise ratios (SNR) for each user, due to unique channel transmittances *η*_*l*_. We note that under statistical variations of estimated noise and transmittance, the information reconciliation parameters will likely change. However, assuming respective *β*_*i*_ and FER_*i*_ per user remain the same, and the most pessimistic channel parameters within Gaussian confidence intervals with *δ* = 10^−10^ failure probability^31^, the total network key rate for untrusted broadcasting reduces to ${K}_{\Sigma }^{{\rm {U}}}=0.4$Mbits/s, while trusted broadcasting can retain a significant rate advantage ${K}_{\Sigma }^{{\rm {T}}}=1.0$ Mbits/s.

**Table 1:** Summary of experimental parameters and CV-QPON protocols performance

User	*η*_*l*_	*V*_*l*_, mSNU	*ε*, mSNU	*β* (%)	FER (%)	*K*^U(TS)^ (kbits/s)
Bob_1_	0.0369	51.24	0.794	90.79	4.5	242.8 (322.6)
Bob_2_	0.0424	52.76	1.558	93.23	43	40.4 (53.1)
Bob_3_	0.0439	55.42	1.23	91.37	22.3	154.3 (208.9)
Bob_4_	0.0397	49.74	0.912	91.5	15.3	227.1 (323.5)
Bob_5_	0.0461	60.14	0.814	91.44	13.6	375.4 (549.2)
Bob_6_	0.0337	53.14	1.002	91.9	21.5	92.01 (121.2)
Bob_7_	0.0398	75.18	1.578	94.8	55.4	20.73 (20.73)
Bob_8_	0.0463	52.66	0.866	90.78	9.5	360.5 (509.6)

*η*_*l*_: channel transmittance, *V*_*l*_: electronics noise, *ε*: excess noise (at the channel output), *β*: information reconciliation efficiency, FER: frame error rate, *K*^U(TS)^: untrusted(trusted) key rate

In the untrusted CV-QPON protocol scenario, Bob_5_ achieved a maximum key rate (*K*^U^) of 375.4 kbit/s, thanks to low excess noise level and high channel transmittance. Notably, under the trusted protocol, the key rate for the same user increased by 46%, underscoring our CV-QPON protocols’ capacity to support simultaneous key generation and enhance network performance. Table 2 compares the performance of access networks with similar architectures and reveals a distinct rate advantage of our work, given that our system has the largest capacity with the highest number of concurrent users. Networks based on DV protocols can be extended to longer distances; however, cannot be efficiently scaled without the use of wavelength-division multiplexing. Under the same efficiency, *β* = 95%, as presumed in ref. ^32^ our QPON can exhibit 731.3 kbits/s (user 5, as shown in Fig. 5a) with double the network capacity.

**Table 2:** Comparison of experimental access networks and expected asymptotic SKR

Reference	Year	Network type	Users/Capacity	Max range (km)	Protocol family	SKR (kbits/s)
^6^	2013	Upstream	2/8	19.9	DV	43.1
^15^	2015	Upstream	2/8	20	DV	33
^45^	2020	Upstream	2/2	12.3	CV	22.19
^14^	2021	Downstream	3/16	21	DV	1.5
^46^	2023	Upstream	3/8	30	CV	0.82
^32^	2024	Downstream	4/4	10	CV	1010
Our work	2024	Downstream	8/8	11	CV	549.2

Adopting finite-size effect analysis of PTP protocols^31,33^, we can establish secure keys with four users (nos. 1, 4, 5, and 8) using the trusted protocol by assuming reconciliation *β* = 96% and protocol failure probability *δ* = 10^−8^.

## Discussion

In CV quantum cryptography, the quantum information is encoded in the quadratures of the electromagnetic field of light and subsequently decoded via coherent detection. This offers the advantage of employing cost-effective, standard telecom components operating at room temperature and a high rate over metropolitan distances compared to discrete-variable protocols^34^. Despite these advantages, the application of CV quantum cryptography has been predominantly confined to point-to-point connections and niche applications in dedicated high-security networks. In our work, we have extended the scope of CV quantum cryptography beyond the point-to-point paradigm to encompass quantum access networks. This expansion has been achieved by introducing continuous-variable quantum passive optical network protocols.

The developed CV-QPON protocol is designed to enable simultaneous key generation- a distinct feature of CV-QPON- and ensure compatibility with conventional downstream access network architectures as well as multiplexing techniques^35–37^. Depending on the trust level assigned to network users, we have outlined the untrusted and trusted CV-QPON protocols, and have experimentally validated the feasibility of these protocols within a CV-QPON setup based on a LLO scheme. Our network facilitates concurrent access to eight users over an 11 km access link, with the potential to scale up the number of users based on the excess noise and the channel loss. We have shown that the trusted protocol significantly enhances the overall network key rate performance and that it is particularly advantageous in scenarios where each user experiences different levels of channel loss.

Unlike the successive quantum state merging protocols^18^, our trusted protocol is compatible with an arbitrary selection of Gaussian states, i.e., both coherent and squeezed states, and offers the flexibility to incorporate the effects of trusted detectors and various side channels^38,39^. A unique feature of this protocol is its strategy for removing information leakage stemming from residual correlations among users and the dissemination of information reconciliation syndromes. This is achieved by establishing a lower bound on the key rate through a hierarchical trust model, obviating the necessity for additional syndrome encryption^27^ while maintaining the secrecy and irreversibility of the protocol’s operations. Compared to time-sharing approaches^6,8^, our CV-QPON protocols demonstrate a definitive advantage in terms of key rate and the capability for concurrent key generation. Nonetheless, there remains room for further enhancements. Theoretically, identifying a CV-QPON protocol that can saturate the channel’s capacity is imperative for achieving optimal performance. This can potentially be accomplished by an alternative multi-user protocol that employs uniform trust assumptions, thereby improving the key rate equally and concurrently for all network users. Addressing the finite-size effects in the current trusted protocol and extending the security proof to encompass discrete modulation are pivotal for enhancing practical implementation and achieving higher rates through the use of high-speed components^34^. Furthermore, reducing excess noise through improved laser technologies and developing of MET-LDPC codes optimized for modulation variance are essential for expanding network capacity and enhancing total network key rates. The aforementioned improvements can be combined with multi-user squeezed-state CV QKD protocol, enabling larger network size and/or secure distance.

In conclusion, our CV-QPON protocol offers a cost-effective, practical solution that seamlessly integrates with standard telecom network infrastructures, thereby facilitating the progression toward a comprehensively interconnected quantum network, such as the European quantum communication infrastructure (EuroQCI).

## Materials and methods

Figure 4 shows the schematic of our CV-QPON network’s experimental setup, where eight users are connected to the provider via an 11 km quantum broadcast channel, incorporating a 1:8 passive optical beam splitter and single-mode fibers (SMF). The provider, Alice, produces an ensemble of coherent states. This process involves two main components: a digital signal processing (DSP) module and an optical module. In the DSP module, the complex amplitude of each coherent state was formed by drawing random numbers from Gaussian distributions, obtained from a vacuum-based quantum random number generator (QRNG)^40^. The quantum symbols, drawn at a rate of 100 MBaud, were upsampled to 1 GSample/s. Subsequently, they were pulse-shaped using a root-raised cosine filter with a roll-off factor of 0.2. The resulting baseband signal was frequency-shifted to center around 170 MHz, aiding in single-sideband modulation. Additionally, a 270 MHz pilot tone was frequency-multiplexed with the passband signal to facilitate carrier phase recovery. The corresponding electrical signal was generated using a digital-to-analog converter (DAC) operating at a sampling rate of 1 GSample/s.

In the optical module, a 1550 nm continuous wave (CW) laser with a linewidth of 100 Hz was used as an optical source. This laser was modulated by an in-phase and quadrature (IQ) modulator driven by the DAC. The IQ modulation was set to operate in optical single-sideband carrier suppression mode. To achieve this, an automatic bias controller (ABC) was used to control the direct current bias voltages applied to the IQ modulator. Following the IQ modulator, a variable optical attenuator (VOA) was used to adjust the modulation variance of the generated thermal state. The quantum signal was then sent to eight receivers through the quantum broadcast channel. Each receiver, on average, experienced a physical loss of ~13.8 dB.

At the receiver ends, each user used coherent detection to measure the incoming coherent states. This involved implementing radio frequency (RF) heterodyne detection, which mixes the quantum signal with a local oscillator (LO) signal in a balanced beamsplitter. The LO was generated by an independent, free-running CW laser, which had a frequency offset of ≈300 MHz relative to Alice’s laser. Due to a limitation of available equipment, all eight receivers shared the same LO, split by another 1:8 beam splitter. A manual polarization controller (PC) was then used to align the polarization of the quantum signal with that of the LO to maximize the visibility of interference fringes. The interference pattern was detected and digitized using a broadband balanced detector (BD) with a bandwidth of ≈250 MHz and a 1 GSample/s analog-to-digital converter (ADC). To emulate the actual scenario of the network setting, each pair of receivers was associated with an independent workstation, each of which was equipped with two-channel ADC cards. These ADCs were clock synchronized with the DAC using a 10 MHz reference clock. The workstations were connected through a local area network, and the entire setup was controlled through a Python-based framework, enabling autonomous modulation and data acquisition.

The users performed three types of measurements: quantum signal, vacuum noise (transmitter’s laser off, LO laser on), and electronic noise (transmitter’s laser off, LO laser off). These measurements were carried out consecutively, and divided into frames, each containing 10^7^ ADC samples. For the modulation variance calibration, a back-to-back measurement was conducted by directly connecting one of the receivers to Alice’s station using a short fiber patchcord. The clearance on the quantum band of each user was, on average, ≈15 dB. Following these measurements, the receivers started the process of recovering their quantum symbols using an offline DSP module.

The DSP technique for quantum symbols recovery involves several steps^28^. First, it applies a whitening filter to remove any correlation between the quantum symbols caused by the detector’s imperfect transfer function. Next, it utilizes a pilot-aided carrier phase recovery, enhanced by employing a machine-learning method based on an unscented Kalman filter^41^. This is followed by temporal synchronization through cross-correlation with predefined reference symbols. The final stages included matched filtering and downsampling to the symbol rate.

Upon completion of the prepare-and-measure phase, users progressed to the subsequent stages of the CV-QPON protocols. The initial step is information reconciliation, a critical process wherein users adopted a multi-dimensional (MD) reconciliation approach^42^. This method relied on multi-edge-type low-density-parity-check (MET-LDPC) error-correcting codes with a rate of 0.01^30^. To enhance reconciliation efficiency, rate-adaptive techniques were integrated^43^. For more detailed information, readers are directed to Supplementary materials.

Subsequently, Alice undertook the task of parameter estimation. Within the framework of an untrusted protocol, Alice constructed covariance matrices for each user to compute the key rate. Conversely, for the trusted broadcast protocol, a covariance matrix describing the overall shared state was reconstructed. Then, the users’ trust sequence was assigned in ascending order based on their key rates obtained from the untrusted broadcast protocol. Specifically, the user with the lowest key rate perceived all others as untrusted, whereas the user with the highest key rate considered all others as trusted, thereby elevating the key rate even more. Such a strategy has been determined to maximize the network key gain. Interestingly, inverting this order enables the user with the lowest key rate to have the most significant enhancement. The final step in both protocols involved implementing privacy amplification^44^.

## Supplementary information

Supplementary Materials for Continuous-variable quantum passive optical network

## Data Availability

All data needed to evaluate the conclusions in this paper are present in the paper and/or the Supplementary Materials.
