# Supplementary material for: Continuous-variable quantum passive optical network
Source: Light Sci Appl. 2024 Oct 16;13:291. doi: 10.1038/s41377-024-01633-9 (PMC11480326; doi:10.1038/s41377-024-01633-9)
Supplement: Supplementary file 1 — Supplementary Materials for Continuous-variable quantum passive optical network [file 41377_2024_1633_MOESM1_ESM.pdf]

Supplementary Materials for

Continuous-variable quantum passive optical network

Adnan A.E. Hajomer[1,*,†], Ivan Derkach[1,2,**,†], Radim Filip[2], Ulrik L. Andersen[1], Vladyslav C. Usenko[2], Tobias Gehring[1,***]

[1]Center for Macroscopic Quantum States (bigQ), Department of Physics, Technical University of Denmark, 2800 Kongens Lyngby, Denmark
[2]Department of Optics, Faculty of Science, Palacky University, 17. listopadu 12, 771 46 Olomouc, Czech Republic
† These authors contributed equally
Corresponding authors: * aaeha@dtu.dk, ** ivan.derkach@upol.cz, *** tobias.gehring@fysik.dtu.dk

1. CONSTRUCTING THE COVARIANCE MATRIX

The secure key rate for a Gaussian CV-QKD protocol can be evaluated using the covariance matrix of the equivalent entanglement-based setup due to the extremality of Gaussian states [S1]. Figure S1(a) shows the entanglement-based version of the prepare-and-measure CV-QPON protocol, where signal and noise sources are purified by a two-mode squeezed vacuum (TMSV) state described with a covariance matrix of the form

$$\Gamma(V) = \begin{bmatrix} V\mathbb{I} & \sqrt{V^2-1}\sigma_z \\ \sqrt{V^2-1}\sigma_z & V\mathbb{I} \end{bmatrix}, \tag{S1}$$

where $\mathbb{I}$ is a 2×2 identity matrix, and Pauli matrix $\sigma_z = \text{diag}\,[1,0,0,-1]$. In this setup, Alice prepares a coherent state for broadcasting by heterodyning one of the modes (A) of the source with covariance matrix $\Gamma(V)$. The initial signal state is given by the covariance matrix $\gamma_{AB} = (S_{1/2}\otimes\mathbb{I})\,[\mathbb{I}\otimes\Gamma(V)]\,(S_{1/2}\otimes\mathbb{I})^T$, with a symplectic transformation $S_{1/2}$ corresponding to a balanced beamsplitter operation. The model of the source can be more involved, accommodating varying levels of modulation for each quadrature, limited squeezing and/or preparation noise[S2, S3].

The broadcasted signal (mode B) propagates through a quantum channel characterized by transmittance η_A and excess noise variance ε_A. The signal mode B is then split into N modes $B_1,\dots B_N$, for each respective user. These modes travel through quantum channels with distinct transmittances $\eta_{B_1}\dots\eta_{B_N}$ and noise variances $\varepsilon_{B_1}\dots\varepsilon_{B_N}$. Typically, the variance of excess noise might vary between the different quadratures. However, in our case, the variances in both quadratures were sufficiently similar, allowing us to simplify our analysis by using their averaged values. In general, Eve's ancillary modes can be correlated, enabling her to extract more information from the broadcasted states during individual measurements. However, we assume all quantum channels are independent, implying that the excess noise is also independent and added after the signal has been split for distribution to users (i.e., $\varepsilon_A = 0$). This is because the influence of noise added before the splitter ε_A would drastically decrease with a growing number of users N. The exploration of more sophisticated attack strategies, which might not adhere to these simplifications, is deferred to future research. After the signal passes through the quantum channels, the resulting covariance matrix, denoted as $\gamma_{AB'}$, contains $2+N$ modes ($A^{x(p)}$, $B_1\dots B_N$):

$$\gamma_{AB'} = \frac{1}{2}\begin{bmatrix} \gamma_{A^xA^p} & \sqrt{2\eta_1(V^2-1)}\varsigma_z & \cdots & -\sqrt{2\eta_N(V^2-1)}\varsigma_z \\ \sqrt{2\eta_N(V^2-1)}\varsigma_z^T & \{(V-1)\eta_1+2+\varepsilon_1\}\mathbb{I} & & -\sqrt{2\eta_1\eta_N}(V-1)\mathbb{I} \\ \vdots & & \ddots & \vdots \\ -\sqrt{2\eta_N(V^2-1)}\varsigma_z^T & -\sqrt{2\eta_1\eta_N}(V-1)\mathbb{I} & \dots & \{(V-1)\eta_N+2+\varepsilon_N\}\mathbb{I} \end{bmatrix}, \text{with } \varsigma_z = \begin{bmatrix} 1 & 0 \\ 0 & -1 \\ 1 & 0 \\ 0 & -1 \end{bmatrix}, \tag{S2}$$

where $\eta_i = \eta_A\eta_{B_i}/N$ is the total transmittance, $\varepsilon_i = \varepsilon_{B_i}$, and $\gamma_{A^xA^p} = \frac{1}{2}\text{diag}\,[(V+1)\mathbb{I},(V-1)\mathbb{I},(V-1)\mathbb{I},(V+1)\mathbb{I},]$.

On the receiving end, each user splits the incoming signal (mode B_i) on a balanced beam splitter into two modes (B_i^x and B_i^p) and measures them with imperfect homodyne detectors in respective quadratures. The imperfect detection is modeled as linear interaction on a beam splitter with transmitance τ. This interaction is accompanied by a thermal noise of variance ν, corresponding to electronic, and purified by a TMSV source (modes D_i and F_i) described by a covariance matrix $\Gamma(V_{D_i})$, with $V_{D_i} = 1+\nu_i/(1-\tau)$. The trusted detection noise can be correlated between quadratures; regardless, it will not contribute to the knowledge of Eve. In scenarios where heterodyne measurements are imbalanced or efficiencies vary by quadrature, the state of Bob$_i$ can be described by a 6 modes covariance matrix containing modes $B_i^{x(p)}$, $D_i^{x(p)}$, and $F_i^{x(p)}$. Here, variances of electronic noise ν_i were averaged over quadratures at each Bob$_i$, similarly as the excess noise ε, and detection efficiencies were common for all users $\tau = \tau_1 = \dots = \tau_N$. Bob$_i$ homodynes the state with variance $V_{B_i^x} = 1 + [(V-1)\eta_i + \varepsilon_i]\,\frac{\tau}{2} + \nu_i$ in the mode B_i^x, and the covariance matrix of modes B_i^x, D_i^x and F_i^x after the interactions can be written as:

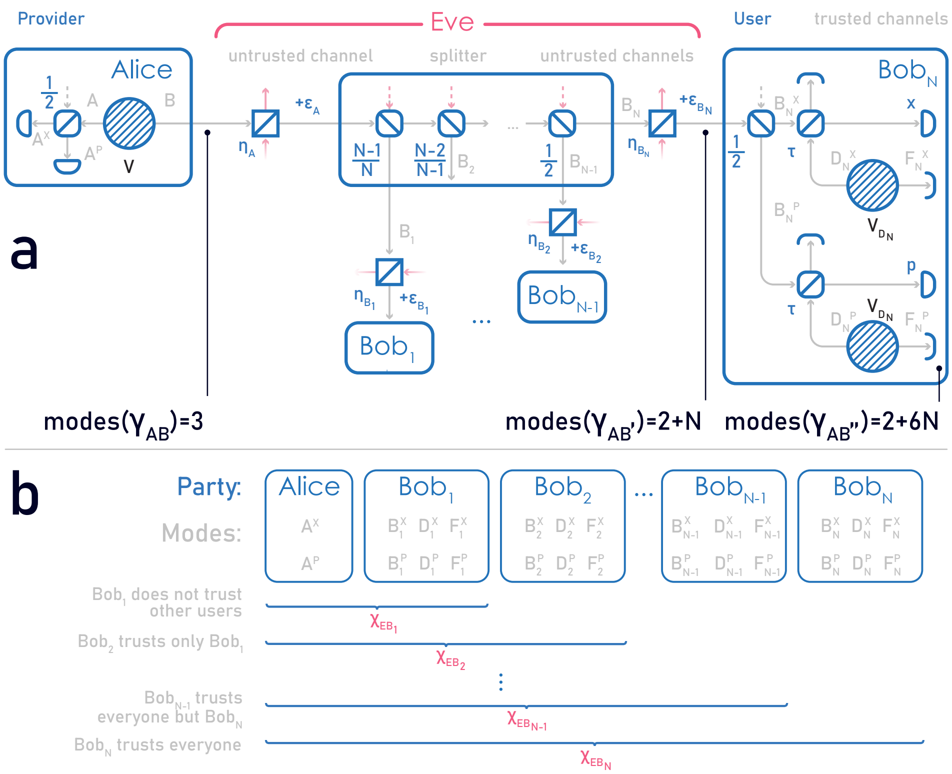

Fig. S1. CV-QPON protocol security analysis and trust assumptions. (a) Broadcasting protocol entanglement-based scheme. (b) Successive user trust in trusted broadcasting protocol.

$$\gamma_{B_i^x D_i^x F_i^x} = \begin{bmatrix} V_{B_i^x}\mathbb{I} & \tau\frac{2\nu_i-(1-\tau)(\varepsilon_i+(V-1)\eta_{B_i})}{2\sqrt{\tau(1-\tau)}}\mathbb{I} & \sqrt{\frac{\nu_i^2+2\nu_i(1-\tau)}{(1-\tau)}}\sigma_z \\ \tau\frac{2\nu_i-(1-\tau)(\varepsilon_i+(V-1)\eta_{B_i})}{2\sqrt{\tau(1-\tau)}}\mathbb{I} & \frac{1-\tau}{2}\left(2+\varepsilon_i+(V-1)\eta_{B_i}\right)+\tau V_{D_i}\mathbb{I} & \sqrt{\tau\nu_i\frac{\nu+2(1-\tau)}{(1-\tau)^2}}\sigma_z \\ \sqrt{\frac{\nu_i^2+2\nu_i(1-\tau)}{(1-\tau)}}\sigma_z & \sqrt{\tau\nu_i\frac{\nu_i+2(1-\tau)}{(1-\tau)^2}}\sigma_z & V_{D_i}\mathbb{I} \end{bmatrix}. \tag{S3}$$

The mode B_i^x is correlated to mode B_i^p as $[\eta_i\varepsilon_i - \eta_{B_i}(V-1)(1-\eta_A)]\sqrt{\frac{\tau}{2(1-\eta_i)\eta_i}}$, and with $C_{i,j}^x = B_j^x$ as $-\frac{\tau}{2}(V-1)\sqrt{\eta_i\eta_j}$. With N users the overall broadcasted state will be contained in up to $2+6N$ modes, with the covariance matrix $\gamma_{AB''}$ that will serve a foundation for the security analysis of network protocols.

2. SECURITY ANALYSIS

The secure key rate between Alice and any Bob$_i$ is determined by Devetak-Winters formula [S4]:

$$K_i(\eta,\varepsilon) = \max\left[0, \beta_i I_{AB_i} - \chi_{EB_i}\right], \tag{S4}$$

where both the mutual information I_{AB_i} and the Holevo bound χ_{EB_i} can be evaluated based on the covariance matrix $\gamma_{AB''}$. The former is the same for time-sharing and broadcasting protocols and is commonly determined by signal-to-noise ratio (SNR) as,

$$I_{AB_i} = \frac{1}{2}\log_2\left[1+\frac{\frac{\eta_i\tau}{2}(V-1)}{1+\nu_i+\varepsilon_i\frac{\tau}{2}}\right]. \tag{S5}$$

The mutual information between users

$$I_{B_iB_j} = \frac{1}{2}\log_2\left[\frac{V_{B_i^x}}{V_{B_i^x}-\frac{(C_{i,j}^x)^2}{V_{B_j^x}}}\right],$$

corresponding to passive state preparation [S5], which quickly deteriorates with increasing number of users or extending distance from the provider.

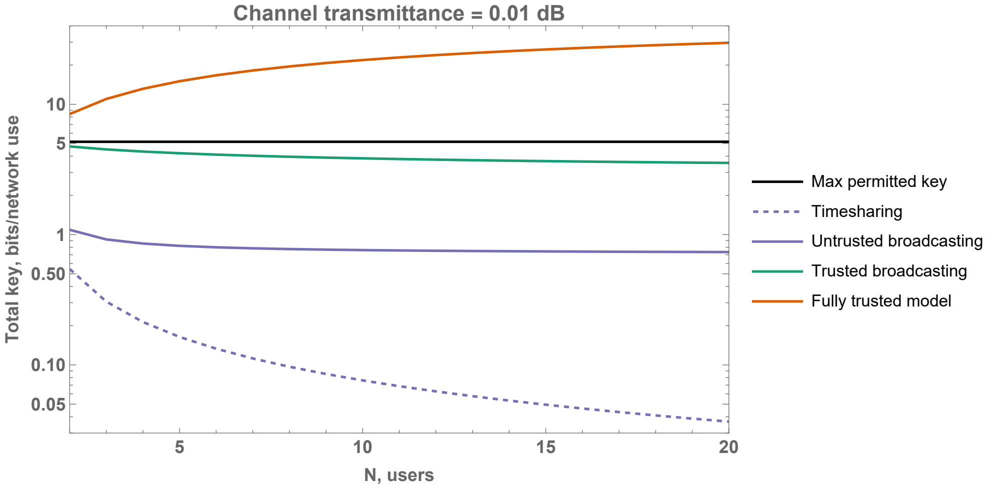

Fig. S2. Total network key comparison between protocols. Noiseless channels $\varepsilon = 0$, squeezing modulation variance $V_{x(p)} = 100$ SNU, perfect reconciliation $\beta = 100\%$ and detectors $\tau = 100\%$, $\nu = 0$.

In asymptotic regime, where collective attacks have been shown to be optimal [S6, S7], Eves' knowledge on the measurement results of the reference user is upper bounded by the Holevo bound $\chi_{EB_i} = S(\rho_E) - S(\rho_{E|B_i})$ [S8]. Eve holds the purification of the shared trusted state ρ_{AB_iC}, with $tr(\rho_{AB_iCE}) = 1$, where state ρ_C corresponds to the part of broadcasted signal distributed to other non-reference users (and respective trusted measurements). Consequently, the bound is simplified to $\chi_{EB_i} = S(\rho_{AB_iC}) - S(\rho_{AC|B_i})$, and can be evaluated based on the re-constructed covariance matrix $\gamma_{AB''}$.

While the sub-systems of Alice ρ_A and reference user ρ_{B_i} (which also holds the purification of relevant trusted noise and loss) are settled, the main difference between the protocols is the degree of trust that is defined by what part of the signal is assigned to sub-system ρ_C (or equivalently to ρ_E). For time-sharing and untrusted broadcast protocols, all users are treated as part of Eve's system, i.e., all parts of the signal not received by the reference user are assumed to be part of ρ_E. In this case, the state ρ_{AB_iC} is described by the covariance matrix containing modes $A_{x(p)}$, $B_i^{x(p)}$, $D_i^{x(p)}$, and $F_i^{x(p)}$. On the other hand, when the particular user (Bob$_j$) is regarded as trusted, modes $B_j^{x(p)}$, $D_j^{x(p)}$ and $F_j^{x(p)}$ are now assigned to the ρ_C sub-system, leading to a decrease in Eve's knowledge. When all users are assumed to be trusted then a full $\gamma_{AB''}$ covariance matrix with $2 + 6N$ modes is used for the evaluation of the Holevo bound. A user operation is identical to the one in PTP protocols, and the reconstruction of the total covariance matrix along with determining individual Holevo bounds is up to the Provider.

A. Comparison of protocols

In both, the time-sharing and untrusted broadcasting protocols, the user's key rate will be the same, potentially achieving the maximum limit set by the PLOB bound [S9], which is determined by the channel transmittance η_i. The main distinction between these protocols lies in their ability to generate keys simultaneously. While the raw keys shared among users are not independent as $I_{B_iB_j} > 0$, privacy amplification processes effectively decouple the keys from Eve, whose state also presumably contains all other users. Consequently, final keys are completely independent among users.

The trusted broadcasting protocol enhances the individual key rate of each user by relying on the faithful operation of network users. As illustrated in Fig. S1(b), each user is allowed to designate a unique subset of other users as trusted. The differences between protocols become apparent in conditions of extreme proximity to the Provider. In Fig. S2 all protocols are compared to a standard point-to-point protocol with a single receiver (i.e. without the splitter $N = 1$) and a channel transmittance of $\eta_A\eta_{B_1} = 0.01$ dB—which sets the benchmark for the highest possible key rate. In scenarios where the number of users N increases, the time-sharing approach experiences a rapid decline in key rate, whereas broadcasting protocols demonstrate resilience in maintaining a level of total key generation regardless of the network size. The signal loss significantly impacts the untrusted protocol, due to considering additional users as part of Eve's state. Conversely, trusting all users can result in underestimating Eve's potential knowledge χ_{EB_i}, given the high correlation among all raw keys; knowledge of one key can offer insights into the others. This suggests that a comprehensive trust approach necessitates additional costs for privacy amplification. By allowing the number of trusted users to vary for each key, the trusted broadcasting protocol effectively circumvents this problem.

Extending the idea to the squeezed-state protocol, we note that the performance of the trusted broadcasting protocol is comparable with the broadcast protocol based on quantum state merging [S10]. The latter regards all users as untrusted when estimating the Holevo bound, but successively increases the joint mutual information between users. Both protocols provide a similar key rate, as shown in Fig. S3, with a minor advantage of the state-merging-based protocol in the absence of trusted detection noise. Such noise effectively decouples Eve not only from a particular user but also from all other users as well. However, this cannot be taken into account during successive state merging as electronic noise has the same effect on mutual information regardless of it being trusted or not.

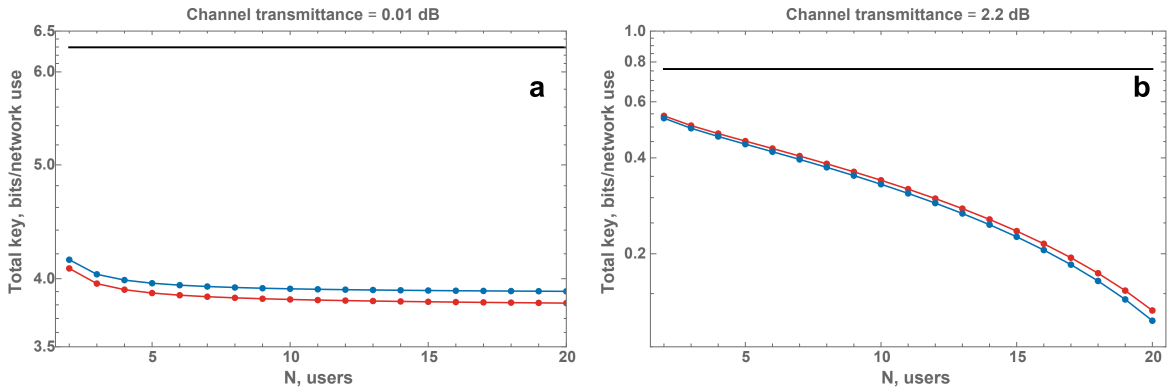

Fig. S3. Total network key comparison between squeezed-state trusted broadcasting (red) and state-merging-based (blue) protocols with maximal permitted key (black). (a) Noiseless channels $\varepsilon = 0$, modulation variance $V_{x(p)} = 100$ SNU, perfect reconciliation $\beta = 100\%$ and detectors $\tau = 100\%$, $\nu = 0$. (b) Channel noise $\varepsilon = 0.5\%$ SNU, modulation variance $V_{x(p)} = 4$ SNU, reconciliation efficiency $\beta = 95\%$, detector efficiency $\tau = 86\%$, electronic noise $\nu = 2\%$ SNU.

Table S1. Performance of adaptive reconciliation protocol. The original MET-LDPC code has a rate $R = 0.01$, with codework length $n = 8.192 \times 10^5$ and information bits $k = 8192$.

User	SNR	p	s	R_{pu}	R_{sh}	β,%	FER,%
Bob_1	0.0077	10000	–	0.0101	–	90.79	4.5
Bob_2	0.0088	130000	–	0.0118	–	93.23	43
Bob_3	0.0091	140000	–	0.0120	–	91.37	22.3
Bob_4	0.0083	70000	–	0.0109	–	91.5	15.3
Bob_5	0.0096	170000	–	0.0126	–	91.44	13.6
Bob_6	0.00708	–	550	–	0.0093	91.9	21.5
Bob_7	0.0082	90000	–	0.0112	–	94.8	55.4
Bob_8	0.0097	170000	–	0.0126	–	90.78	9.5

B. Information reconciliation

The information reconciliation (IR) process utilized a multi-dimensional (MD) reconciliation scheme, employing a multi-edge-type low-density-parity-check (MET-LDPC) code with a rate of 0.01 and codeword length $n = 8.192 \times 10^5$ [S11]. This code is theoretically designed to operate at a SNR of 0.007. However, in the context of CV-QPON, users experience varying channel transmittances, which results in differing received SNRs. Consequently, the efficiency of the IR exhibits significant variation across different users.

To enhance the performance of IR for all users within CV-QPON, we adopted a rate-adaptive reconciliation protocol. This approach allows for the flexible adjustment of the MET-LDPC code's rate according to the specific received SNR of each user. Through the application of puncturing and shortening techniques [S12], we can effectively modify the code rate either by increasing or decreasing it. The adjusted code rate is defined as, $R_{punc} = k/(n-p)$, for puncturing and $R_{sh} = (k-s)/(n-s)$ for shortening, where k represents the number of information bites and p and s are the lengths of puncturing and shortening, respectively. Table S1 shows the modified rates along with the corresponding efficiency and frame error rate (FER) for each user. Most users deployed puncturing as their received SNR was above 0.007. On the other hand, despite operating at an SNR of approximately 0.007, Bob_6 had to employ a shortening strategy. This is because, for the code with finite length, the code rate needs to be strictly smaller than the channel capacity.

The selection of puncturing and shortening lengths cannot be made arbitrarily due to the inherent trade-off between FER performance and efficiency β [S13–S15]. Increasing the code rate through puncturing—by removing information—elevates the FER because the likelihood of incorrect frame decoding rises [S16]. Conversely, shortening the code has the opposite effect. To implement these techniques, we developed a framework capable of processing 5.9 million symbols per second. This level of performance was facilitated by utilizing an NVIDIA GeForce RTX 2060 Mobile graphics processing unit (GPU) with the system consuming 2.5 gigabytes of memory.

C. Correlation of users

To demonstrate our CV-QPON protocols' ability to enable simultaneous key generation among users, we examined correlations of the users' measurement results. Figure S4 shows the mutual information (MI) between Bob_1 and other users, revealing a low correlation, as indicated by small MI values (highlighted by a black square in the figure inset). The rather small correlations are largely due to the

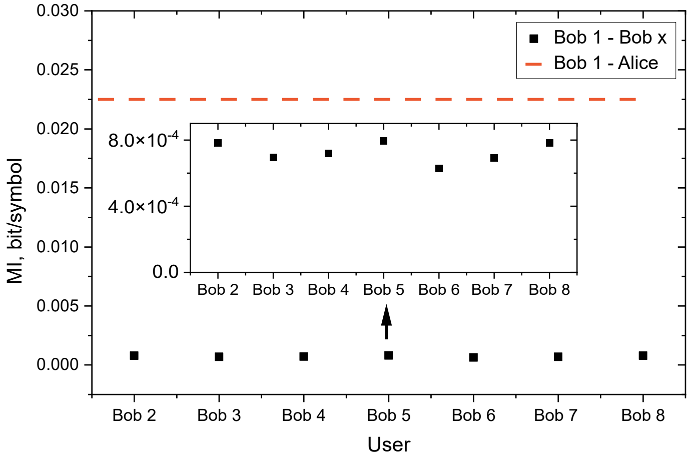

Fig. S4. Analysis of user correlations within the network. Experimentally obtained mutual information (MI) between Bob_1 and other network users compared to the mutual information between Bob_1 and the provider, Alice. The inset shows the MI with a different scale.

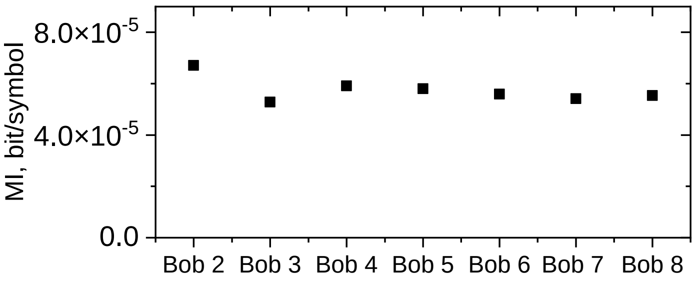

Fig. S5. Analysis of noise correlation measured by different users. The excess noise of Bob_1 is considered as a reference for mutual information measurement.

independently measured quantum noise by each user, with a dominant component being phase noise [S14]:

$$\xi_{\text{PN}} = 2\eta V_{\text{M}} \left(1 - e^{\frac{-\sigma_{\text{PN}}}{2}}\right), \tag{S6}$$

where ξ_{PN} is the excess noise due to the phase noise, η is the channel transmittance, V_M represents modulation variance, and σ_{PN} is the variance of the phase noise. In contrast, the MI between Alice and Bob_1 is substantially higher, by two orders of magnitude compared to other users, as represented by the red dashed line. Therefore, implementing reverse reconciliation [S17] allows Alice and each user to gain an information advantage, thus facilitating the concurrent reconciliation of independent keys, which is a distinctive aspect of the CV-QPON protocols.

To conduct the analysis of correlations between noise measured by different users, we infer the total noise variables based on Bob's measurement as,

$$\xi_l^{x(p)} = B_l^{x(p)} - g \times A^{x(p)}. \tag{S7}$$

Here, g represents a scaling factor, which can be calculated as $\text{Cov}(B_l^{x(p)}, A^{x(p)})/\text{Var}(A^{x(p)})$, where Cov and Var are covariance and variance, respectively. Figure S5 displays the mutual information between the noise measured by Bob_1 and another user. The mutual information between the noise measurements across users is an order of magnitude lower than observed among users. This residual correlation can be attributed to the finite-size effects of these Gaussian variables. Thereby, the noise measured by users is largely independent, and any observed correlation among users can mainly be attributed to Alice's modulation, as depicted in Fig S4. These results support our assumption of noise independence, aligning closely with practical implementations.

REFERENCES

[S1] Michael M Wolf, Geza Giedke, and J Ignacio Cirac. Extremality of gaussian quantum states. *Physical review letters*, 96(8):080502, 2006.

[S2] Vladyslav C Usenko and Radim Filip. Squeezed-state quantum key distribution upon imperfect reconciliation. *New Journal of Physics*, 13(11):113007, 2011.

[S3] Ivan Derkach, Vladyslav C Usenko, and Radim Filip. Continuous-variable quantum key distribution with a leakage from state preparation. *Physical Review A*, 96(6):062309, 2017.

[S4] Igor Devetak and Andreas Winter. Distillation of secret key and entanglement from quantum states. *Proceedings of the Royal Society A: Mathematical, Physical and engineering sciences*, 461(2053):207–235, 2005.

[S5] Bing Qi, Philip G Evans, and Warren P Grice. Passive state preparation in the gaussian-modulated coherent-states quantum key distribution. *Physical Review A*, 97(1):012317, 2018.

[S6] Anthony Leverrier. Composable security proof for continuous-variable quantum key distribution with coherent states. *Physical review letters*, 114(7):070501, 2015.

[S7] Anthony Leverrier. Security of continuous-variable quantum key distribution via a gaussian de finetti reduction. *Physical review letters*, 118(20):200501, 2017.

[S8] Alexander S Holevo and Reinhard F Werner. Evaluating capacities of bosonic gaussian channels. *Physical Review A*, 63(3):032312, 2001.

[S9] Stefano Pirandola, Riccardo Laurenza, Carlo Ottaviani, and Leonardo Banchi. Fundamental limits of repeaterless quantum communications. *Nature communications*, 8(1):15043, 2017.

[S10] Masahiro Takeoka, Kaushik P Seshadreesan, and Mark M Wilde. Unconstrained capacities of quantum key distribution and entanglement distillation for pure-loss bosonic broadcast channels. *Physical review letters*, 119(15):150501, 2017.

[S11] Hossein Mani, Tobias Gehring, Philipp Grabenweger, Bernhard Ömer, Christoph Pacher, and Ulrik Lund Andersen. Multiedge-type low-density parity-check codes for continuous-variable quantum key distribution. *Physical Review A*, 103(6):062419, 2021.

[S12] Xiangyu Wang, Yichen Zhang, Song Yu, Bingjie Xu, Zhengyu Li, and Hong Guo. Efficient rate-adaptive reconciliation for continuous-variable quantum key distribution. *Quantum Information & Computation*, 17(13-14):1123–1134, 2017.

[S13] Adnan AE Hajomer, Nitin Jain, Hossein Mani, Hou-Man Chin, Ulrik L Andersen, and Tobias Gehring. Modulation leakage-free continuous-variable quantum key distribution. *npj Quantum Information*, 8(1):136, 2022.

[S14] Adnan AE Hajomer, Ivan Derkach, Nitin Jain, Hou-Man Chin, Ulrik L Andersen, and Tobias Gehring. Long-distance continuous-variable quantum key distribution over 100-km fiber with local local oscillator. *Science Advances*, 10(1):eadi9474, 2024.

[S15] Nitin Jain, Hou-Man Chin, Hossein Mani, Cosmo Lupo, Dino Solar Nikolic, Arne Kordts, Stefano Pirandola, Thomas Brochmann Pedersen, Matthias Kolb, Bernhard Ömer, et al. Practical continuous-variable quantum key distribution with composable security. *Nature communications*, 13(1):4740, 2022.

[S16] Jesus Martinez-Mateo, David Elkouss, and Vicente Martin. Blind reconciliation. *Quantum Information & Computation*, 12(9-10):791–812, 2012.

[S17] Frédéric Grosshans, Gilles Van Assche, Jérôme Wenger, Rosa Brouri, Nicolas J Cerf, and Philippe Grangier. Quantum key distribution using gaussian-modulated coherent states. *Nature*, 421(6920):238–241, 2003.
